# Effects of propranolol and isoproterenol on infantile hemangioma endothelial cells *in vitro*

**DOI:** 10.3892/etm.2014.1780

**Published:** 2014-06-12

**Authors:** YALIN ZHU, AERZIGULI TUERXUN, YAN HUI, PARIDE ABLIZ

**Affiliations:** 1 Department of Dermatology, The First Affiliated Hospital of Xinjiang Medical University, Urumqi, Xinjiang 830054, P.R. China; 2 Medical Research Center, The First Affiliated Hospital of Xinjiang Medical University, Urumqi, Xinjiang 830054, P.R. China

**Keywords:** infantile hemangioma, endothelial cells, propranolol, isoproterenol, β-adrenergic receptor

## Abstract

The aim of the present study was to investigate the effects of propranolol and isoproterenol on the growth curve of infantile hemangioma endothelial cells (IHECs) *in vitro* and determine the functions of the β-adrenergic receptor in the pathogenesis of infantile hemangioma. IHECs were divided into three groups: The control group, the propranolol group (PG) and the isoproterenol group (IG). The PG and IG were administered with high, medium and low concentrations of the corresponding drugs. The cell growth in each group was determined using the MTT assay. A high propranolol concentration resulted in the inhibition of cell growth. By comparison, isoproterenol promoted cell growth. Within a specific time-frame (72–96 h), high drug concentrations (20 μg/ml) elicited strong effects on the cells. At certain concentrations, propranolol inhibited cell growth once the proliferation stage of IHECs had been affected for a specific length of time, whereas isoproterenol yielded opposite results. The β-adrenergic receptor elicits an important effect in the pathogenesis of infantile hemangioma.

## Introduction

At present, the nomenclature of skin vascular tissue tumors remains inconsistent, particularly in vascular tissue tumors. Different classifications have also been used unsatisfactorily. In 1996, the International Society of Vascular Anomalies divided the vascular birthmarks into two categories: Vascular tumors and vascular malformations; infantile hemangioma, for example, is classified as a vascular tumor (1,2). Infantile hemangioma is one of the most common benign tumors in infants and young children; these tumors are superficially located in the head and neck area (3). A few cases have also been observed in the mucosa, muscle, bone tissue and internal organs, which are affected in terms of appearance and function. In extreme cases, these superficial tumors threaten the life of the patient. Different theories, including the heredity and gene mutation theory, the placental chorionic cell ectopia theory, the endothelial progenitor cell or stem cell theory and the angiogenesis imbalance theory (4–14), have been proposed regarding the pathogenesis of hemangioma; however, no decisive conclusion has been obtained to date. Propranolol is a nonselective β-adrenergic receptor agonist. In 2008, a study proposed that propranolol elicits significant adverse effects on hemangioma (15); thus, propranolol has been increasingly used as the first-line treatment against hemangioma. Therefore, the functions of the β-adrenergic receptor in the pathogenesis of vascular tumors have attracted increased attention. Isoproterenol is a β-adrenergic receptor antagonist. A previous study demonstrated that isoproterenol promoted angiogenesis *in vitro*, indicating that the β-adrenergic receptor is important in the occurrence and development of vascular tumors (16).

In the present study, specific concentrations of propranolol and isoproterenol were used to compare the effects on infantile hemangioma endothelial cells (IHECs) in an *in vitro* cultivation environment of IHECs. This experiment was conducted to further investigate the functions of the β-adrenergic receptor in the development and progression of vascular tumors.

## Subjects and methods

### Subjects

A hemangioma specimen was resected clinically from a proliferating hemangioma on the forehead of a nine-month-old female patient. This study was conducted in accordance with the Declaration of Helsinki and with approval from the Ethics Committee of The First Affiliated Hospital of Xinjiang Medical University (Urumqi, China). Written informed consent was obtained from the guardian of the infant.

### Primary cultivation of IHECs

The resected hemangioma specimen was immediately placed and stored in RPMI-1640 serum-free medium and promptly transferred to a laboratory laminar flow cabinet. Following the removal of the supernatant, the specimen was placed in a sterile Petri dish and rinsed with double-antibody phosphate-buffered saline (PBS; HyClone Laboratories, Inc., South Logan, UT, USA) twice. The dish was replaced and washed three time with PBS. Specimen trimming was performed in the Petri dish to remove the epidermis and the connective tissues, prior to the specimen being cut into 1×1 mm sections and washed twice with PBS. A 0.25% trypsin (HyClone Laboratories, Inc.) solution was used to digest the cells for 3–4 h at 37°C with constant agitation. The RPMI-1640 medium, containing 20% fetal bovine serum (Gibco^®^-BRL, Grand Island, NY, USA), was then added to terminate digestion. The mixture was filtered and centrifuged (179 × g for 5 min) and the supernatant was subsequently discarded. Another portion of the RPMI-1640 medium was used to resuspend the precipitate. The supernatant was centrifuged (179 × g, 5 min) and discarded. Endothelial cell growth medium 2 (EGM-2), containing vascular endothelial growth factor (VEGF), hydrocortisone, ascorbic acid, human alkaline fibroblast growth factor B, human insulin-like growth factor and epidermal growth factor (Lonza Ltd., Basel, Switzerland), was added to the mixture, which was then transferred into a 25-cm^2^ flask and placed in an incubator with CO_2_ at 37°C. After 24 h, the cells adhered to the flask walls. The medium was changed at intervals of 2–3 days. Generation passage was performed when the cells covered 70–80% of the flask bottom. All of the procedures were completed under sterile conditions in the laminar flow cabinet.

### Identification of IHECs

The primary cells were obtained and digested with 0.25% trypsin solution. The digested cells were then centrifuged (179 × g, 5 min) and EGM-2 was used to resuspend the precipitate. The resulting suspension was transferred to the slide of a 10-cm dish and developed at 37°C and with CO_2_. After 24 h, the supernatant was discarded. The slide was washed three times with PBS, fixed with 4% paraformaldehyde for 20 min and washed a further three times with PBS; blocking solution was subsequently added to the slide, which was kept at room temperature for 30 min. The slide was then washed with PBS, and primary rabbit anti-human von Willebrand factor (vwf) polyclonal antibody (Wuhan Boster Biological Technology, Ltd., Wuhan, China) was added to the slide. The cells were subsequently cultivated overnight at 4°C. In the blank control group, PBS was used as the primary antibody. Following overnight cultivation, the slide was washed three times with PBS. The secondary antibody, polymerized horseradish peroxidase (HRP)-labeled anti-rabbit immunoglobulin G (IgG) (Wuhan Boster Biological Technology, Ltd.), was added to the slide, and the cells were cultivated for 30 min at 37°C. The specimen was stained using a Diaminobenzidine Chromogenic Staining kit (Beijing Sequoia Jinqiao Biological Technology Co., Ltd., Beijing, China) for 2 min. Staining was terminated with distilled water, and the specimen was observed under a microscope.

A second experimental set was prepared using the same procedure as above; however, rabbit anti-human VEGF receptor 2 (VEGFR-2) antibody (Wuhan Boster Biological Technology, Ltd.) was instead used as the primary antibody, and polymerized HRP-labeled anti-rabbit IgG antibody was used as the secondary antibody. In the blank control group, PBS was used as the primary antibody.

### Determination of cell growth curves using the MTT assay

The second-generation cells were spread evenly on a 96-well plate at a concentration 2×10^4^ cells per well. EGM-2 (~200 μl) was added and the cells were incubated for 24 h. The medium was subsequently changed and eight cells from each row of wells were observed daily. Approximately 20 μl MTT solution (5 mg/ml; Sigma, St. Louis, MO, USA) was added daily into each well and cultivation was continued for 4 h, prior to the supernatant being discarded. Approximately 150 μl dimethyl sulfoxide (DMSO; Sigma) was then added, and the system was agitated for 10 min. A microplate reader (Thermo Fisher Scientific, Inc., Rockford, IL, USA) was used to determine the absorbance of each well at a wavelength of 490 nm for 10 consecutive days. In the cell growth curve, time was set as the abscissa and the average absorbance was set as the ordinate.

### Effect of propranolol on cell growth curves

Propranolol (Tianjin Lisheng Pharmaceutical Co., Ltd., Tianjin, China) was dissolved in DMSO and then diluted with EGM-2 to prepare working solutions at three concentrations: 10, 15 and 20 μg/ml. The final DMSO concentration was 0.16%. Following the preparation, the solutions were filtered with a 0.22-μm filter and sub-packaged.

The second-generation cells were obtained for digestion, centrifugation and counting. These cells were evenly spread on a 96-well plate at a cell concentration of 2×10^4^ cells per well; 200 μl EGM-2 was added and the cells were incubated for 24 h. Following adhesion of the cells to the walls, the medium was replaced with the 10, 15 and 20 μg/ml working solutions, respectively. Blank EGM-2 (without propranolol) and 0.16% DMSO-containing EGM-2 were used as the controls, with each treatment replicated four times. The cells were cultured in a CO_2_ incubator, and the absorbance was determined using the MTT assay at 24, 48, 72 and 96 h; simultaneously, the morphological changes were observed and recorded. The non-parametric test of the multiple-sample-related measurements was performed to compare the difference in absorbance between each concentration and the control group. The average absorbance curve of the cells at each concentration was plotted, with time as the abscissa and average absorbance as the ordinate.

### Effects of isoproterenol on cell growth curves

The effects of isoproterenol were observed following the procedures used to determine the effects of propranolol; however, isoproterenol was used instead of propranolol.

### Statistical analysis

The non-parametric test of the multiple-sample-related measurements used in the data analysis was performed using SPSS 17.0 (SPSS, Inc., Chicago, IL, USA).

## Results

### Cultivation of IHECs

**Figure 1:**
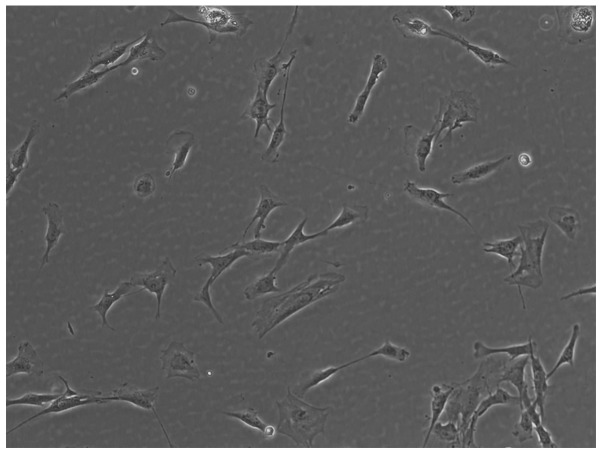
Primary cultivation of infantile hemangioma endothelial cells (magnification, ×10).

After the 24-h primary cultivation of IHECs, the cells gradually adhered to the walls, appearing round to polygonal in shape. Between days 2 and 3, the cell number gradually increased, and between days 4 and 5 the cells, half of which appeared polygonal, gradually fused. Between days 8 and 9, the cells gradually became fusiform and funicular, and blood vessel-like structures were visible in certain regions. Generation passage was performed when the cells occupied 80% of the bottom of the container (Fig. 1).

### Identification of IHECs

**Figure 2:**
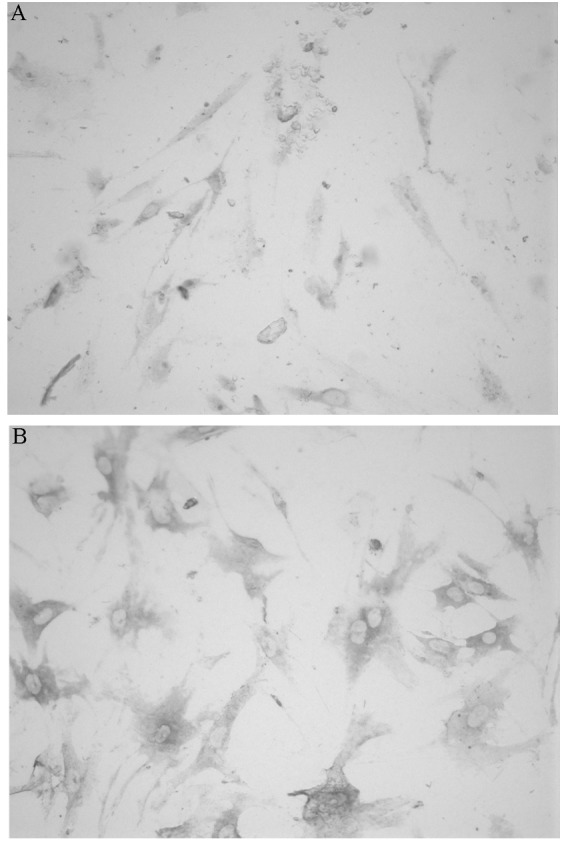
(A) Immunohistochemistry of von Willebrand factor (magnification, ×10). (B) Immunohistochemical detection of vascular endothelial growth factor receptor 2 (magnification, ×10).

Upon conducting immunohistochemistry, it was observed that the cells were polygonal and spindle-shaped with brownish-yellow-stained cytoplasm; the nucleus remained unstained, indicating a positive result. These results were consistent with those in previous studies (17,18), in which vwf was present in the cytoplasm; therefore, it was confirmed that these cells were IHECs (Fig. 2A). A similar staining method was used to determine the presence of VEGFR-2 in the cytoplasm, yielding the same positive results as the previous experiment and confirming that the cells were IHECs (Fig. 2B).

### Determination of the growth curve of the IHECs

**Figure 3:**
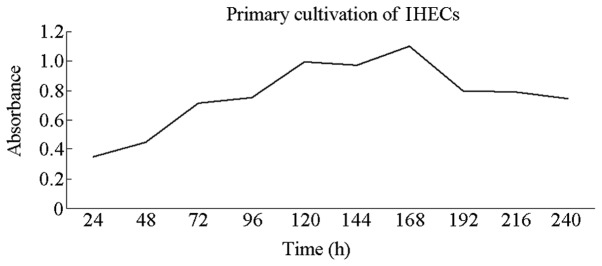
Growth curve of the second-generation IHECs (MTT assay). IHECs, infantile hemangioma endothelial cells.

The MTT assay was used to determine the IHEC growth curves. The results indicated a slow increase in the average absorbance of the cells on days 1 and 2. This parameter rapidly increased between days 3 and 5 but slightly decreased at day 6. On days 7 and 8, absorbance increased, prior to decreasing and then reached a plateau. Absorbance decreased gradually between days 9 and 10 (Fig. 3). This result was consistent with those from other studies (17,18).

### Effect of propranolol on IHECs

**Figure 4:**
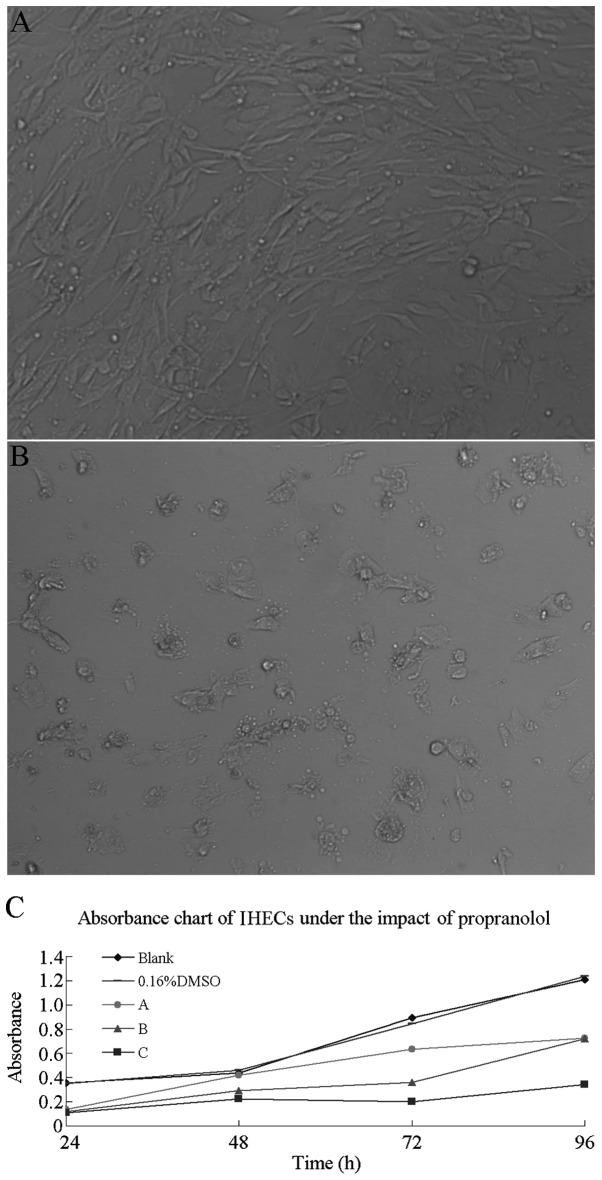
(A) IHECs prior to the co-cultivation with propranolol (96-well plate; magnification, ×10). (B) IHECs 96 h after the action of 20 μg/ml propranolol (magnification, ×10). (C) Absorbance chart of IHECs under the impact of propranolol. Concentrations of propranolol used in groups A–C were 10, 15 and 20 μg/ml, respectively. IHECs, infantile hemangioma endothelial cells; DMSO, dimethyl sulfoxide.

In the *in vitro* culture environment, the absorbance of IHECs was measured using the MTT assay following cultivation in 10, 15 and 20 μg/ml propanol working solution, respectively, for 24, 48, 72 and 96 h. SPSS 17.0 (SPSS, Inc.) software was used for the statistical analysis of the absorbance at each concentration. The results showed no significant difference in the average absorbance of the IHECs among the blank, the DMSO and the three propranolol concentration groups in the 24- to 48-h time-frame (P>0.05). However, a difference in absorbance was observed in the 72-to 96-h time-frame. The inter-group comparison revealed that while no significant differences were observed among the control, the DMSO and the 10 and 15 μg/ml propranolol groups, a significant difference was identified between the 20 μg/ml group and the blank group (P<0.05). A change was also observed in the cell morphology: The IHECs in each well initially proliferated and then adhered to the walls 24 h before the medium was replaced (Fig. 4A). Comparing this result with the conditions in the well after 96 h and the replaced medium, it was found that the morphological characteristics of IHECs had changed (Fig. 4B). In particular, the IHECs appeared round or almost round, the cellular space increased and the cell number was low. The stacked line chart of IHEC absorbance under different concentrations of propranolol is shown in Fig. 4C.

### Effect of isoproterenol on IHECs

**Figure 5:**
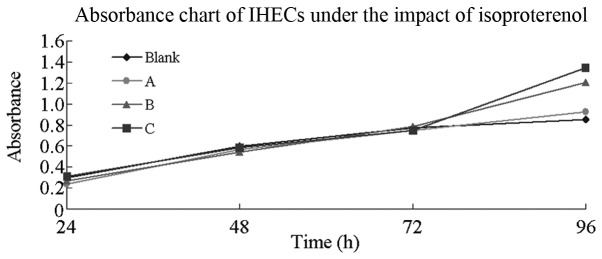
Absorbance chart of IHECs under the impacts of isoproterenol. Concentrations of isoproterenol used in groups A–C were 10, 15 and 20 μg/ml, respectively. IHECs, infantile hemangioma endothelial cells.

In the *in vitro* culture environment, the average absorbance of the IHECs was measured using the MTT assay following cultivation in 5, 10 and 20 μg/ml isoproterenol working fluid, respectively, for 24, 48, 72 and 96 h. Isoproterenol-free medium was used as the blank control group. No significant differences were found in the average absorbance of the IHECs among the groups in the 24-to 72-h time-frame (P>0.05); however, an absorbance difference was observed at 96 h among the blank group and the isoproterenol-containing groups. The inter-group comparison revealed that a high isoproterenol concentration corresponded to a high absorbance (P<0.05). The stacked line chart of IHEC absorbance under different concentrations of isoproterenol is shown in Fig. 5.

## Discussion

Vascular tumors, including infantile hemangioma, delayed pyogenic granuloma and other rare vascular tumors occurring during infancy and early childhood, are characterized by a proliferation of endothelial cells, while vascular malformations are abnormalities of vascular morphogenesis. Vascular tumors can readily occur in the neonatal period; however, unlike infantile hemangioma, vascular malformations do not grow rapidly nor regress spontaneously in the first year following birth (19).

Infantile hemangioma can be divided into the proliferation period (three to 12 months old), the regression period (one to three years old) and the regression completion period (three to seven years old) (20). Although infantile hemangioma in certain patients undergoes self-regression, hemangioma in the proliferation period reproduces rapidly. This condition can affect the function of important surface organs and internal vital organs and can potentially be life-threatening (19).

The present study targeted infantile hemangioma in the proliferation period (nine months old), and a surgically resected specimen was obtained for the primary cultivation of the IHECs in EGM-2. A previous study on the pathogenesis of hemangioma indicated that growth factors, including VEGFs, fibroblast growth factors and human insulin-like growth factors, have an important function in the formation of vascular tumors (21). The medium used in this study contained various growth factors and selectively promoted the growth of IHECs. In the primary cultivation of IHECs at the proliferation period, normal medium is not able to satisfy the requirements of cell growth, and the medium containing specific cytokines is more suitable. The growth curve of the IHECs shows that the cells grew slowly between days 1 and 2, and that growth increased rapidly between days 3 and 5, decreased slightly at day 6 and increased again between days 7 and 8. A plateau was ultimately reached and growth decreased between days 9 and 10.

Since the discovery of propranolol for the treatment of hemangioma, propranolol has been widely used on affected children and achieved favorable results (22,23). Although the therapeutic mechanism remains unclear, the involvement of the β-adrenergic receptor has been considered. In the present study, two drugs, mutually antagonistic towards each other, were used to investigate the functions of the β-adrenergic receptor in the development of vascular tumors.

Following the intervention with different propranolol-concentration mediums, the results revealed that the low-concentration groups (10 and 15 μg/ml) had no significant adverse effects on the IHECs. In the high-concentration group (20 μg/ml), absorbance decreased between the 72- and 96-h time-points compared with the other groups, indicating that cell growth was inhibited. The morphological observations also showed that cell growth was significantly limited following propranolol action. The growth curves showed that the average absorbance in the high-concentration group was decreased compared with that in the blank control group, indicating that cell growth was suppressed by propranolol. The degree of suppression corresponded to the drug concentration.

Isoproterenol is a non-selective β-adrenergic receptor agonist that exhibits a mutually antagonistic effect with propranolol. The present study identified that, following culture for 24–72 h with a certain concentration of isoproterenol-containing medium, negligible effects were observed in the IHECs of each group. In addition, no significant differences were observed in the absorbance among the groups. After 96 h, a high concentration of isoproterenol corresponded to a high absorbance in each group, indicating that isoproterenol elicited stimulating effects on cell growth. In contrast to the effects of propranolol on the IHECs, isoproterenol elicited a stimulating effect on the IHECs, with an activity degree that was proportional to the concentration.

The present study revealed that propranolol elicits a positive therapeutic effect on infantile hemangioma; however, the mechanism remains unclear. Considering different theories, including the cytokine, signal transduction and receptor theories, the mechanism involved in such effects has yet to be determined. This study used two mutually antagonistic drugs to act on the β-adrenergic receptor. The results showed the contrasting effects of these drugs on IHECs. The results further indicated that the β-adrenergic receptor plays a role in infantile hemangiomas and that the therapeutic effect of propranolol was largely induced via the β-adrenergic receptor. However, further investigation is required to determine the exact mechanism.

## References

[1] Enjolras O, Mulliken JB (1997). Vascular tumors and vascular malformation (new issuses). Adv Dermatol.

[2] Mulliken J, Enjolras O (2004). Congenital hemangiomas and infantile hemangioma: missing links. J Am Acad Dermatol.

[3] Cahill AM, Nijs EL (2011). Pediatric vascular malformations: pathophysiology, diagnosis, and the role of interventional radiology. Cardiovasc Intervent Radiol.

[4] Itinteang T, Tan ST, Guthrie S, Tan CE, McIntyre BC, Brasch HD, Day DJ (2011). A placental chorionic villous mesenchymal core cellular origin for infantile haemangioma. J Clin Pathol.

[5] Régnier S, Dupin N, Le Danff C, Wassef M, Enjolras O, Aractingi S (2007). Endothelial cells in infantile haemangiomas originate from the child and not from the mother (a fluorescence in situ hybridization-based study). Br J Dermatol.

[6] Zhang GY, Yi CG, Li X, Liang ZQ, Wang RX, Liu DE, Zhang LM, Meng CY, Guo SZ (2008). Proliferation hemangiomas formation through dual mechanism of vascular endothelial growth factor mediated endothelial progenitor cells proliferation and mobilization through matrix metalloproteinases 9. Med Hypotheses.

[7] Takahashi K, Mulliken JB, Kozakewich HP, Rogers RA, Folkman J, Ezekowitz RA (1994). Cellular markers that distinguish the phases of hemangioma during infancy and childhood. J Clin Invest.

[8] Peng Q, Matsuda T, Hirst SJ (2004). Signaling pathways regulating interleukin-13-stimulated chemokine release from airway smooth muscle. Am J Respir Crit Care Med.

[9] Queto T, Vasconcelos ZF, Luz RA (2011). G-CSF suppresses allergic pulmonary inflammation, downmodulating cytokine, chemokine and eosinophil production. Life Sci.

[10] Gordillo GM, Onat D, Stockinger M, Roy S, Atalay M, Beck FM, Sen CK (2004). A key angiogenic role of monocyte chemoattractant protein-l in hemangioendothelioma proliferation. Am J Physiol Cell Physiol.

[11] Eum SY, Maghni K, Hamid Q, Eidelman DH, Campbell H, Isogai S, Martin JG (2003). Inhibition of allergic airways inflammation and airway hyperresponsiveness in mice by dexamethasone: role of eosinophils, IL-5, eotaxin, and IL-13. J Allergy Clin Immunol.

[12] North PE, Waner M, Mizeracki A, Mihm MC (2000). GLUT-1: a newly discovered immunohistochemical marker for juvenile hemangiomas. Hum Pathol.

[13] Bauland CG, Smit JM, Bartelink LR, Zondervan HA, Spauwen PH (2010). Hemangioma in the newborn: increased incidence after chorionic villus sampling. Prenat Diagn.

[14] Boye E, Yu Y, Paranya G, Mulliken JB, Olsen BR, Bischoff J (2001). Clonality and altered behavior of endothelial cells from hemangiomas. J Clin Invest.

[15] Léauté-Labrèze C, Dumas de la Roque E, Hubiche T, Boralevi F, Thambo JB, Taïeb A (2008). Propanolol for severe hemangiomas of infancy. N Engl J Med.

[16] Ji Y, Chen S, Li K, Xiao X, Zheng S, Xu T (2013). The role of β-adrenergic receptor signaling in the proliferation of hemangioma-derived endothelial cells. Cell Div.

[17] Ma G, Lin XX, Jin YB (2008). Isolation, culture and identification of endothelial cells in infantile hemangioma. Zhonghua Zheng Xing Wai Ke Za Zhi.

[18] Ma G, Lin XX, Jin YB (2008). Culture and cryopreservation of endothelial cells in vitro in infantile hemangioma. Zhonghua Zheng Xing Wai Ke Za Zhi.

[19] Bolognia JL, Jorizzo JL, Rapini RP (2012). Dermatology.

[20] Léauté-Labrèze C, Prey S, Ezzedine K (2011). Infantile haemangioma: part I. Pathophysiology, epidemiology, clinical features, life cycle and associated structural abnormalities. J Eur Acad Dermatol Venereol.

[21] Takahashi K, Mulliken JB, Kozakewich HP, Rogers RA, Folkman J, Ezekowitz RA (1994). Cellular markers that distinguish the phases of hemangioma during infancy and childhood. J Clin Invest.

[22] Sans V, de la Roque ED, Berge J, Grenier N (2009). Propranolol for severe infantile hemangiomas: follow-up report. Pediatrics.

[23] Sommers Smith SK, Smith DM (2002). Beta blockade induces apoptosis in cultured capillary endothelial cells. In Vitro Cell Dev Biol Anim.

